# Cost-effectiveness analysis of cabazitaxel for metastatic castration resistant prostate cancer after docetaxel and androgen-signaling-targeted inhibitor resistance

**DOI:** 10.1186/s12885-020-07754-9

**Published:** 2021-01-07

**Authors:** Peng-Fei Zhang, Dan Xie, Qiu Li

**Affiliations:** 1 Department of Medical Oncology, Cancer Center, West China Hospital, Sichuan University, Chengdu, China; 2 West China Biomedical Big Data Center, West China Hospital/West China School of Medicine, Sichuan University, Chengdu, China; 3 Prenatal Diagnosis Center, Department of Obstetrics and Gynecology, West China Second University Hospital, Sichuan University, Chengdu, China; 4 Key Laboratory of Birth Defects and Related Diseases of Women and Children (Sichuan University), Ministry of Education, Chengdu, China

**Keywords:** Cabazitaxel, Abiraterone, Enzalutamide, Docetaxel, Cost-effectiveness, Prostate cancer

## Abstract

**Background:**

The aim of our study was to evaluate the cost-effectiveness of cabazitaxel versus abiraterone or enzalutamide in patients with metastatic castration-resistant prostate cancer (mCRPC) previously treated with docetaxel who had progression within 12 months while receiving an alternative inhibitor (abiraterone or enzalutamide) from a US payer’s perspective.

**Methods:**

To conduct the cost-effectiveness analysis, a Markov decision model was established. Three health states (progression-free survival (PFS), progressive disease (PD) and death) were included, and the incremental cost-effectiveness ratio (ICER) was regarded as the primary endpoint. The willingness-to-pay (WTP) threshold was set at $100,000.00/quality-adjusted life year (QALY), and discounted rates were set at 3% annually. Efficacy data were derived from the CARD trial and Weibull distribution curves were modeled to fit the survival curves. The robustness of the analysis was tested with a series of one-way sensitivity analyses and probabilistic sensitivity analyses.

**Results:**

Overall, the incremental effectiveness and cost of cabazitaxel versus androgen-signaling-targeted inhibitors (ASTIs) were 0.16 QALYs and $49,487.03, respectively, which yielded an ICER of $309,293.94/QALY. Our model was mostly sensitive to the duration of PFS in the cabazitaxel group, cost of cabazitaxel and utility of the PFS state. At a WTP threshold of $100,000.00/QALY, cabazitaxel was the dominant strategy in 0% of the simulations.

**Conclusions:**

Cabazitaxel is unlikely to be a cost-effective treatment option compared with ASTIs in patients with mCRPC previously treated with docetaxel who had progression within 12 months while receiving ASTIs.

## Background

Prostate cancer represents the second most commonly diagnosed cancer and the fifth leading cause of cancer-related deaths in men worldwide [1]. Previously, the standard first-line treatment was androgen- deprivation therapy (ADT), which could be achieved by bilateral orchiectomy or luteinizing hormone-releasing hormone (LHRH) agonists/antagonists [2]. However, most patients with advanced prostate cancer will become refractory to ADT, which denotes metastatic castration-resistant prostate cancer (mCRPC) [3]. Several novel drugs, such as docetaxel, androgen-signaling-targeted-inhibitors (ASTIs) and sipuleucel-T, have been demonstrated to prolong survival in patients with mCRPC [4–8]. Recently, ASTIs, such as abiraterone and enzalutamide, have also been demonstrated to be effective in the treatment of metastatic hormone-sensitive prostate cancer in combination with ADT [9–12]. Based on these studies, ASTIs (abiraterone or enzalutamide) and docetaxel are frequently used in patients with prostate cancer in earlier stages, and most of the patients are likely to receive both ASTIs and docetaxel, in either order.

Despite the effectiveness of these regimens, most patients will still become refractory. Once this occurs, a switch to a subsequent ASTI or the use of cabazitaxel has been the standard medical practice. Recently, the efficacy of poly (ADP-ribose) polymerase (PARP) inhibitors, such as olaparib and rucaparib, has also been investigated in patients with mCRPC that harbored alterations in the DNA-damage-repair genes [13, 14]. These studies showed PARP inhibitors could achieve longer survival than a second-generation androgen-deprivation therapy (abiraterone or enzalutamide) in these patients. However, although all these regimens have showed efficacy in the treatment of post-docetaxel mCRPC patients, evidence for the treatment sequence of these drugs is lacking. Based on previous studies, patients may become resistant to abiraterone or enzalutamide after disease progression on previous ASTI treatment [15–17]. Moreover, some evidence also suggests partial cross-resistance between ASTIs and docetaxel [18]. Thus, it would be important to investigate which treatment regimen is superior in patients previously treated with docetaxel and ASTIs. Recently, the results of the CARD trial, which evaluated the efficacy and safety of cabazitaxel versus abiraterone or enzalutamide in patients with mCRPC previously treated with docetaxel who had progression within 12 months while receiving ASTIs, were published [19]. Cabazitaxel significantly prolonged the median imaging-based progression-free survival (PFS) (8.0 months versus 3.7 months) and median overall survival (OS) (13.6 months versus 11.0 months) compared with ASTIs, indicating that cabazitaxel is a more favorable treatment option for patients with mCRPC previously treated with docetaxel and ASTIs.

Despite the benefit achieved by cabazitaxel, the high cost of the treatment may significantly increase healthcare expenditures. Given the heavy healthcare burden worldwide currently, it is crucial to determine which regimen is with better efficiency and pharmacoeconomic profile [20, 21]. The aim of the study was to evaluate the cost-effectiveness of cabazitaxel versus ASTIs (abiraterone or enzalutamide) in patients with mCRPC previously treated with docetaxel who had progression within 12 months while receiving ASTIs from a US payer’s perspective.

## Methods

### Model structure

A Markov decision model was established to evaluate the cost-effectiveness of cabazitaxel versus ASTIs in patients with mCRPC previously treated with docetaxel who had progression within 12 months while receiving an alternative inhibitor (abiraterone or enzalutamide). PFS, progressive disease (PD) and death were defined as the three mutually exclusive health states in the model. All participants were assumed to enter the model in the PFS state, at the end of each cycle, the patients could stay in the starting health state or transition to the PD state or death. Once in the PD state, patients could remain in that state or transition to death at the end of each cycle [22]. Health utilities for the PFS state and PD state were derived from previously published literature [22]. Adverse events (AEs) in the model were chosen if they occurred with high frequency (> 5%), were expensive to treat or substantively affected quality of life (Table 1). Regardless of the influence of AEs, the health utility values in the model were assumed to be invariable. Quality-adjusted life-years (QALYs) and cost were measured, and the incremental cost-effectiveness ratio (ICER) of cabazitaxel versus ASTIs was regarded as the primary endpoint in the study. The willingness-to-pay (WTP) threshold in the analysis was set at $100,000.00/QALY [23, 24]. Effectiveness and cost outcomes were discounted at a 3% annual rate in the model [23, 25]. The time horizon of the model was defined as 10 years, and the Markov cycle length in the model was 3 weeks, which is consistent with the length of the treatment periods. This study was approved by the Ethics Committee of our hospital. The model was developed and tested using the R statistical environment (version 3.6.1; R Development Core Team, Vienna, Austria) and TreeAge software (TreeAge, Williamstown, MA, USA, 2011).

**Table 1:** Key clinical data in the model

Parameters	Cabazitaxel	ASTI	Reference
**Weibull parameters**			
**Scale (λ) for PFS**	0.034827	0.069115	[19]
**Shape (γ) for PFS**	1.486146	1.468545	[19]
**Scale (λ) for OS**	0.007118	0.007698	[19]
**Shape (γ) for OS**	1.676228	1.867383	[19]
**Survival**			
**Median OS (range), month**	13.6 (11.5–17.5)	11.0 (9.2–12.9)	[19]
**Median imaging PFS (range), month**	8.0 (5.7–9.2)	3.7 (2.8–5.1)	[19]
**Median PFS (range), month**	4.4 (3.6–5.4)	2.7 (2.4–2.8)	[19]
**Treatment**			
**Median treatment duration (range), month**	22 (3–88)	12.5 (2–141)	[19]
**Median treatment cycles**	7 (1–29)	4 (1–45)	[19]
**Probability of AEs (grade 3/4)**			
**Musculoskeletal pain or discomfort**	0.016 (0.013–0.019)	0.056 (0.045–0.067)	[19]
**Renal disorder**	0.032 (0.026–0.038)	0.081 (0.065–0.097)	[19]
**Anemia**	0.080 (0.064–0.096)	0.048 (0.038–0.058)	[19]
**Leukopenia**	0.320 (0.256–0.384)	0.016 (0.013–0.019)	[19]
**Neutropenia**	0.447 (0.358–0.536)	0.032 (0.026–0.038)	[19]
**Utility**			
**PFS**	0.617 (0.494–0.740)	0.617 (0.494–0.740)	[22]
**PD**	0.370 (0.296–0.444)	0.370 (0.296–0.444)	[22]

*ASTI* androgen-signaling-targeted inhibitor, *PFS* progression-free survival, *OS* overall survival, *AEs* adverse events

### Patients and treatments

A cohort population reflecting the participants of the CARD trial was modeled in the study [19]. Patients who were confirmed as metastatic prostate cancer histologically and had previously been treated with three or more cycles of docetaxel, and had previously had disease progression during 12 months of treatment with an androgen-signaling–targeted inhibitor, were included. Cabazitaxel was administered intravenously at a dose of 25 mg per square meter of body surface area (BSA) every 3 weeks. Meanwhile, these patients also received 10 mg oral prednisone daily. Based on the results of the CARD trial, of the 124 patients who received an ASTI, 58 received abiraterone and 66 received enzalutamide. Abiraterone was given at 1000 mg orally once daily, and oral prednisone was given at 5 mg twice daily, while enzalutamide was administered orally at a dose of 160 mg once daily every 3 weeks. Abiraterone was given to enzalutamide-resistant patients, and enzalutamide was given to patients who failed previous abiraterone treatment.

### Efficacy inputs

**Fig. 1:**
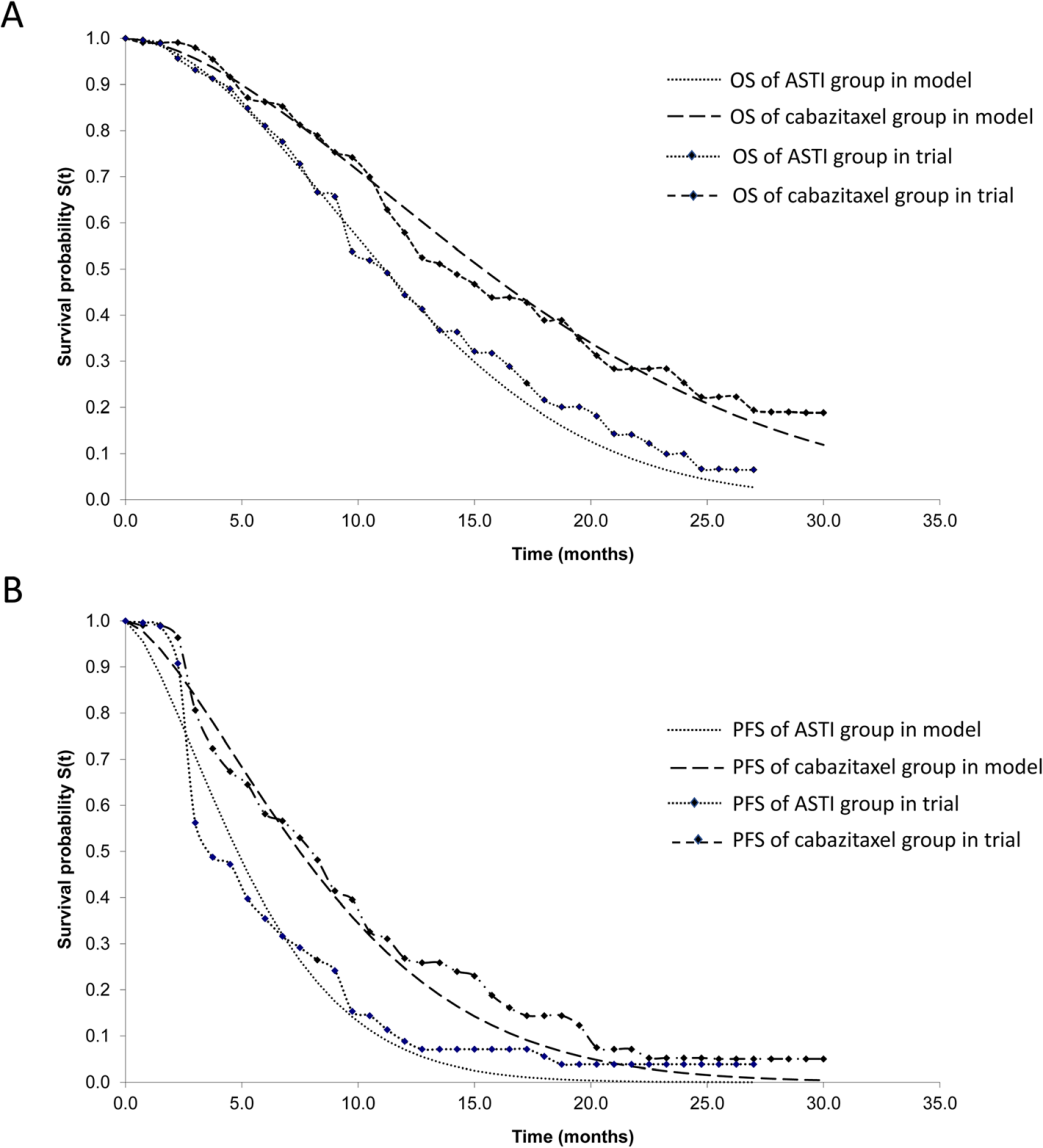
Estimated Weibull survival curves for cabazitaxel group (**a**) and ASTI group (**b**). PFS, progression-free survival; OS, overall survival; ASTI, androgen-signaling-targeted inhibitor

Efficacy data in the model were derived from the CARD trial, and the information was used to estimate the transition probabilities between health states. Survival data points were extracted from the survival curves using a plot digitizer software (DigitizeIt, version 2.0, Braunschweig, Germany, www.digitizeit.de). Weibull distribution curves were modeled to fit the survival curves in the CARD trial. The fitting Weibull parameters (scale (λ) and shape (γ)) are presented in Table 1, and the calibration curves are shown in Fig. 1. The probabilities of progression and death were estimated from the curves as described by a previous study [26].

### Cost and resource data

The cost in the model was estimated from the perspective of US payers, and the costs estimated in the trial are presented in Table 2. The costs of drugs, drug administration, follow-up (reflecting the frequency of drug administration), tests, AE-related treatments and best supportive care (BSC) were included in the model. The unit prices of drugs were obtained from the average whole prices from Red Book Online®, an online resource that lists drug product pricing of medications in the US. To calculate the dose of cabazitaxel, a patient with a height of 176 cm and weight of 88.9 kg (BSA of 2.1 m^2^) was assumed [30]. The unit cost of tests, drug administration, follow-up, tests, AE-related treatments and BSC were retrieved from the Centers for Medicare & Medicaid (CMS) clinical laboratory fee schedule files and previously published literature [27–30]. The median number of treatment cycles was 7 in the cabazitaxel group and 4 in the ASTI group. AE-related cost was calculated by multiplying the incidence of AEs by the cost of managing these AEs per event.

**Table 2:** Cost parameters input in the model

Parameters	Value (range)	Resource
Cabazitaxel (60 mg)	13,170.31 (10,536.248–15,804.384)	RED BOOK
Abiraterone (250 mg)	95.26 (76.208–114.312)	RED BOOK
Enzalutamide (40 mg)	115.486 (92.389–138.583)	RED BOOK
Prednisone (10 mg)	0.53 (0.424–0.636)	RED BOOK
Prednisone (5 mg)	0.39 (0.312–0.468)	RED BOOK
Administration per unit CPT:96365	74.16 (59.328–88.992)	CMS
Musculoskeletal pain or discomfort per event	364.8 (291.84–437.76)	[26], 2015
Renal disorder per event	5966.67 (4773.336–7160.004)	[27], 2015
Anemia per event	1881 (1826–1910)	[27], 2015
Leukopenia per event	3066 (2758–3384)	[27], 2015
Neutropenia per event	3066 (2758–3384)	[27], 2015
Laboratory tests per event	76 (68–84)	[27], 2015
Chest/Abdomen/Pelvis CT per event	828 (598–1083)	[27], 2015
PSA per event	25 (20–30)	[28], 2015
Bone scanning per event	253.46 (202.768–304.152)	[28], 2015
Cost of supportive care per cycle	1213 (987–1438)	[29], 2018
Routine follow-up of patients per unit	422 (348.1–495.8)	[29], 2018

*CT* Computed Tomography, *PSA* prostate specific antigen

### Sensitivity analysis

The robustness of the analysis was tested with a series of one-way sensitivity analyses on several parameters. In the one-way sensitivity analyses, parameters ranged between − 20 and + 20%, and the results of the one-way sensitivity analyses are presented as tornado diagrams. Moreover, probabilistic sensitivity analyses were also performed based on Monte Carlo simulations with 1000 iterations. Gamma distributions were used for cost inputs and beta distributions were used for the probabilities of AEs and utility values. The results of the analyses are shown as cost-effectiveness acceptability curves.

## Results

### Base case analysis

Over a 10-year time lifetime horizon, cabazitaxel produced an effectiveness of 0.57 QALYs per patient at a total cost of $105,169.70, while ASTIs achieved an effectiveness of 0.41 QALYs per patient at a cost of $55,682.67. Overall, the incremental effectiveness and cost of cabazitaxel versus ASTIs were 0.16 QALYs and $49,487.03 per patient, respectively, which yielded an ICER of $309,293.94/QALY gained with cabazitaxel versus ASTIs (Table 3).

**Table 3:** Base case results of the model

Model outcomes	Cabazitaxel	ASTI
**Cost ($)**		
Costs in PFS	94,672.44	43,831.50
Costs in PD	10,497.26	11,851.17
Total costs	105,169.70	55,682.67
Incremental costs	49,487.03	–
**Effectiveness (QALYs)**		
QALYs in PFS	0.40	0.19
QALYs in PD	0.17	0.22
Total effectiveness	0.57	0.41
Incremental effectiveness	0.16	–
**ICER ($/QALY)**	309,293.94	

*ASTI* androgen-signaling-targeted inhibitor, *PFS* progression-free survival, *OS* overall survival, *QALYs* quality-adjusted life years, *IECR* incremental cost-effectiveness ratio

### Sensitivity analyses

**Fig. 2:**
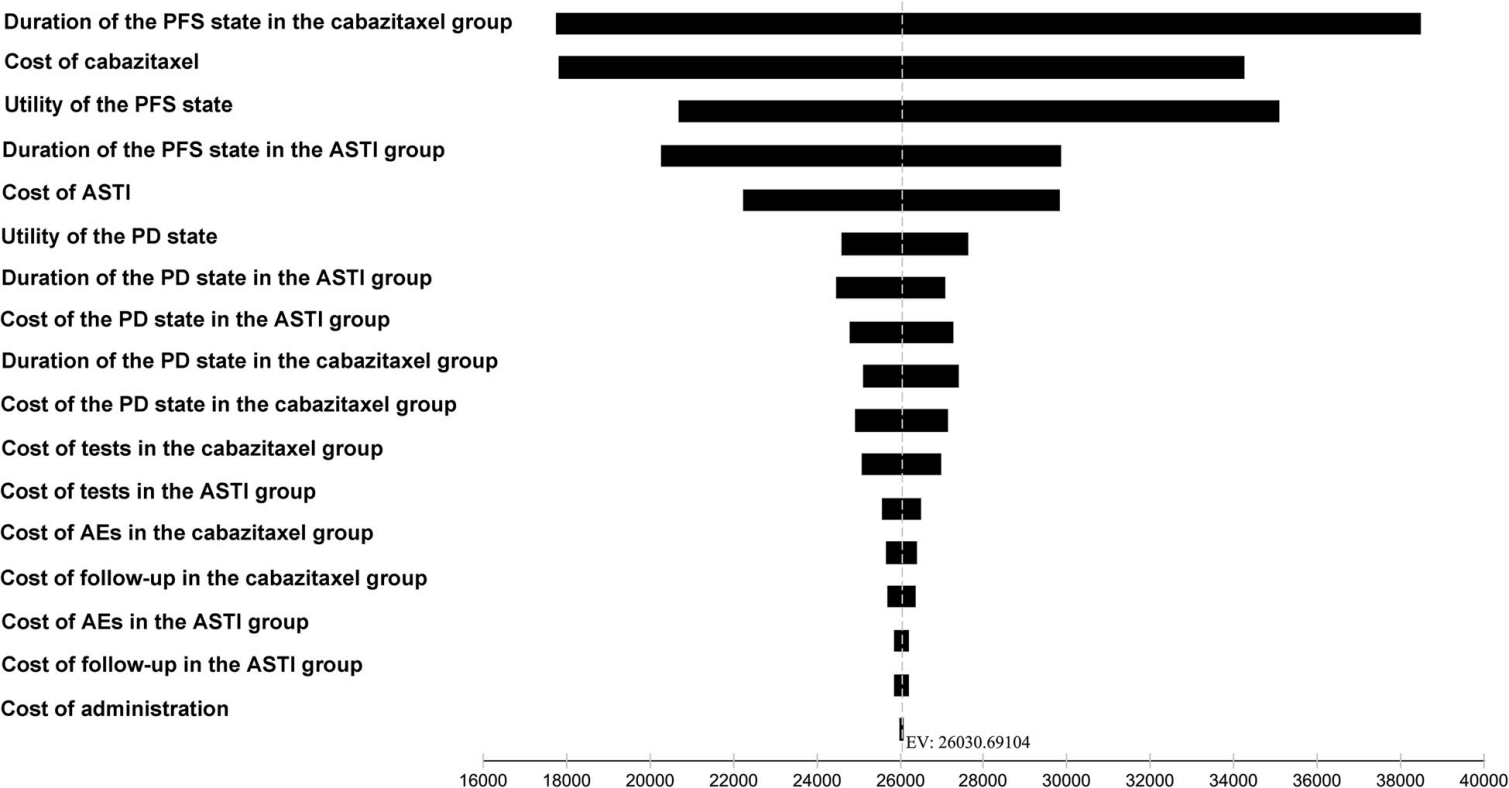
Tornado diagram for one-way sensitivity analyses. PFS, progression-free survival; PD, progressive disease; AEs, adverse events; ICER, incremental cost-effectiveness ratio; ASTI, androgen-signaling-targeted inhibitor

**Fig. 3:**
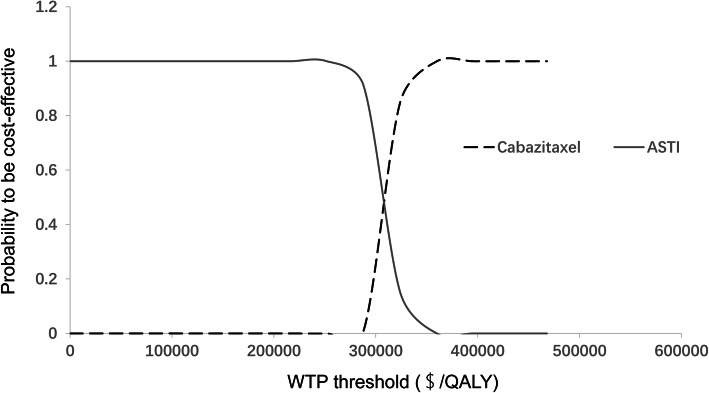
The cost-effectiveness acceptability curves for probabilistic sensitivity analyses. QALY, quality-adjusted life year; WTP, willingness-to-pay

The results of the one-way sensitivity analyses are presented in Fig. 2. The top 3 parameters that mostly impacted the ICER were duration of PFS in the cabazitaxel group, cost of cabazitaxel and utility of the PFS state. The ICER was also sensitive to the duration of PFS in the ASTI group and cost of ASTIs. Other parameters, such as cost of drug administration and cost of follow-up, had little impact on the results of our analysis. In the probabilistic sensitivity analyses, the proportion of cabazitaxel as the more cost-effective strategy compared with ASTIs was 0% when the WTP threshold was set at $100,000.00/QALY (Fig. 3).

## Discussion

Prostate cancer remains one of the most common malignancies in men worldwide. In recent years, ADT combined with docetaxel, abiraterone, or enzalutamide, have been demonstrated to be effective treatment options for patients with metastatic hormone-sensitive prostate cancer. However, for patients with mCRPC previously treated with docetaxel who had progression within 12 months while receiving an alternative inhibitor (abiraterone or enzalutamide), evidence for the treatment sequence of drugs is lacking. In the CARD trial, cabazitaxel was demonstrated to significantly prolong survivalcompared with the ASTIs, which suggested cabazitaxel as the optimal treatment option for patients with mCRPC who had been previously treated with docetaxel and ASTIs.

In this study, however, we evaluated the benefit of cabazitaxel versus ASTIs in patients with mCRPC previously treated with docetaxel and alternative inhibitors from a pharmacoeconomic perspective. Overall, cabazitaxel achieved more effectiveness than ASTIs (0.57 QALYs versus 0.41 QALYs); however, the cost in the cabazitaxel group was much higher than that in the ASTI group ($105,169.70 versus $55,682.67). The ICER of cabazitaxel versus ASTIs was $309,293.94/QALY. Based on the WTP threshold of $100,000.00/QALY, cabazitaxel might not be a cost-effective treatment option compared with ASTIs.

In the sensitivity analyses, the parameters having the greatest influence on the ICER were the duration of PFS in the cabazitaxel group, cost of cabazitaxel, utility of the PFS state, the duration of PFS in the ASTI group and cost of ASTIs. Thus, selecting the subgroup of patients with the best survival benefit of cabazitaxel is essential to improve the pharmacoeconomic profile of cabazitaxel versus ASTIs. On the other hand, cost of cabazitaxel and cost of ASTIs were two other key parameters, which was likely because of their high prices. In addition, the median duration of treatment was longer in patients receiving cabazitaxel than in those receiving ASTIs (22.0 weeks vs. 12.5 weeks), and the median number of treatment cycles received was also higher in patients receiving cabazitaxel than in those receiving ASTIs (7 vs. 4), which reflected the higher disease progression rates (in 43.7 and 71.0% of the patients, respectively) and poorer outcomes in the ASTI group.

To the best of our knowledge, this is the first economic analysis to evaluate the cost-effectiveness of cabazitaxel versus ASTIs for patients with mCRPC who had been previously treated with docetaxel and alternative androgen-signaling-targeted agents using a Markov model. In a recent study, investigators compared abiraterone plus prednisone (ABI+PRD), cabazitaxel plus prednisone (CAB+PRD) and enzalutamide (ENZ) for visceral metastatic CRPC post-docetaxel therapy resistance development [22]. The LYs and QALYs were 1.20 and 0.58, respectively, for ABI+PRD, 1.48 and 0.56 for CAB+PRD, and 1.58 and 0.79 for ENZ. The total treatment cost was: $115,433 for ABI+PRD, $85,337 for CAB+PRD and $109,213 for ENZ. CAB+PRD and ENZ were superior to ABI+PRD due to the higher LYs gained. This analysis found that ENZ provided greater LYs and QALYs than both ABI+PRD and CAB+PRD at a lower cost than ABI+PRD, but at a higher cost than CAB+PRD. For patients with visceral mCRPC after docetaxel therapy resistance, ENZ was cost-effective 92% of the time with a WTP threshold of $100,000/QALY. The cost and effectiveness of the cabazitaxel group in the above study were similar to those in our study; however, the cost and effectiveness in the abiraterone and enzalutamide groups were much higher than those in our analysis. This could be explained by the fact that the above study was conducted based on the results of three studies, the TROPIC trial that compared CAB+PRD to mitoxantrone plus prednisone [5], the COU-AA-301 trial that compared ABI+PRD to placebo plus prednisone [7], and the AFFIRM trial that compared ENZ to placebo plus prednisone [8]. The median durations of treatment were 8 months and 8.3 months in the groups that received abiraterone acetate plus prednisone and enzalutamide, respectively, which were higher than the median number of treatment cycles in the CARD trial. However, the number of treatment cycles of patients in the cabazitaxel group in the TROPIC trial was similar to the number of cycles in the CARD trial.

In addition to cabazitaxel, other novel drugs, were also investigated in the setting of metastatic castration-resistant prostate cancer who had disease progression while receiving a new hormonal agent (e.g., enzalutamide or abiraterone). mCRPC is a heterogeneous disease and a series of genomic aberrations have been identified, which included deleterious aberrations in genes involved in repairing DNA damage [31, 32]. Germline or somatic mutations in DNA damage repair (DDR) genes, such as BRCA2, CHEK2, ATM, RAD51D, BRCA1, and PALB2, have been described in around 20–25% of advanced prostate cancer patients, which involved in aggressive disease and poor outcomes in these patients [31]. However, they also might be therapeutic targets. Recently, the effect of a series of PARP inhibitors has been investigated in patients with mCRPC that harbored DDR mutations. In the PROfound trial, de Bono et al. assessed the efficacy and safety of olaparib in patients with mCRPC harboring DDR mutations who had disease progression while receiving a new hormonal agent (e.g., enzalutamide or abiraterone). Olaparib was associated with longer PFS (median, 7.4 months vs. 3.6 months; HR, 0.34; 95% CI, 0.25 to 0.47; *P*< 0.001) than either enzalutamide or abiraterone [13]. In another phase II study, Abida et al. evaluated the effect of rucaparib for the treatment of men with mCRPC associated with a deleterious alteration in BRCA or other DDR gene who have progressed after next-generation AR-directed therapy and a taxane-based chemotherapy. Rucaparib was demonstrated to be associated with good antitumor activity in patients with mCRPC and a deleterious BRCA alteration [14]. Although the phase 3 TRITON3 trial (ClinicalTrials.gov number, NCT02975934), which evaluates rucaparib 600 mg BID vs physician’s choice of abiraterone, enzalutamide, or docetaxel in patients with mCRPC and a deleterious germline or somatic *BRCA1*, *BRCA2*, or *ATM* mutation, is going on. We predict that rucaparib will also benefit men with mCRPC harboring BRCA1 or BRCA2 alterations than abiraterone, enzalutamide, or docetaxel. These studies provide evidences that PARP inhibitors might be another promising option for patients with mCRPC that harbored DDR mutations. However, in the CARD trial, no detailed data of patients with mCRPC that harbored DDR mutations were reported. As cabazitaxel and PARP inhibitors all showed superior efficacy than subsequent ASTIs, it is interesting to compare the efficacy and cost-effectiveness of these two options in men with mCRPC that harbored DDR mutations in the future.

The limitations of this study should be addressed. First, the analysis was performed based on data from the CARD trial, which might not fully reflect the status in the real-world. Thus, a cost-effectiveness analysis based on real-world data should be performed when the data are available. Second, the analysis included AEs that were relatively frequent (> 5%), expensive to treat or substantively affected quality of life. The costs of grade 1–2 AEs and AEs with low frequency were not included in the study. Fortunately, the results of the one-way sensitivity analyses demonstrated that the economic results were not sensitive to AE-related parameters. Third, the utility scores in the analysis were derived from previously published literature, which may also undermine the robustness of our results.

## Conclusions

In summary, we established a Markov model to evaluate the cost-effectiveness of cabazitaxel versus ASTIs in patients with mCRPC previously treated with docetaxel who had progression within 12 months while receiving ASTIs from the US payer’s perspective. Based on the results of the study, cabazitaxel is unlikely to be a cost-effective treatment option compared with ASTIs in patients with mCRPC previously treated with docetaxel who had progression within 12 months while receiving the ASTIs.

## Data Availability

The data generated during this study are available from the corresponding author on reasonable request.

## Abbreviations

ADT: Androgen deprivation therapy
LHRH: Luteinizing hormone-releasing hormone
mCRPC: Metastatic castration-resistant prostate cancer
ASTIs: Androgen-signaling-targeted-inhibitors
PFS: Progression-free survival
OS: Overall survival
PD: Progressive disease
AEs: Adverse events
QALYs: Quality-adjusted life-years
ICER: Incremental cost-effectiveness ratio
WTP: Willingness-to-pay
BSA: Body surface area
BSC: Best supportive care
